# Cervical Spine Evaluation by Telephone and Video Visit

**DOI:** 10.7759/cureus.19741

**Published:** 2021-11-19

**Authors:** George Pujalte, Jayson R Loeffert, Tais G. O Bertasi, Raphael A. O Bertasi, Therese F Anderson, Stephan M Esser, Carolina S Paredes-Molina, Shirley A Albano-Aluquin

**Affiliations:** 1 Department of Family Medicine, Mayo Clinic, Jacksonville, USA; 2 Department of Family and Community Medicine, Penn State College of Medicine, Hershey, USA; 3 Department of Orthopaedics and Rehabilitation, Penn State College of Medicine, Hershey, USA; 4 Internal Medicine, Mount Sinai Morningside West, New York, USA; 5 Department of Orthopedics, Southeast Orthopedic Specialists, Jacksonville, USA; 6 Department of Rheumatology, Penn State College of Medicine, Hershey, USA

**Keywords:** cervical spine pain, telemedicine, neck pain, virtual primary care, telehealth

## Abstract

As telecommunication technologies advance, efforts are being made to mitigate direct patient contact in the COVID-19 pandemic due to the risk of contagion. The ability to host telephone and video visits within patient portals within health care institutions will only become increasingly valuable. Neck pain, a common complaint seen in primary care clinics, is well-suited to telemedicine evaluation, as related etiologies are often comparatively straightforward. A good assessment of the cervical spine by telephone or video is possible with the right knowledge and practice.

The purpose of this article is to propose questions and maneuvers that can be used to evaluate the cervical spine via telephone or video, as well as likely diagnoses that can be reached through these.

Phone and video evaluation of the cervical spine can result in valuable data regarding symmetry, range of motion, functional movement patterns, modified strength testing, and provocative testing. The skill set necessary to do telephone and video visits should be included in the curriculum of physician learners.

## Introduction and background

Neck pain is among the most frequent complaints of patients seen in primary care clinics [1]. There are a myriad of possible diagnoses, but the common differentials include myofascial pain, degenerative disc disease, and muscle spasms [2]. These complaints often necessitate timely treatment, but evaluation of patients can become difficult during a pandemic. Fortunately, the technology needed for this type of evaluation already exists. During the coronavirus (COVID-19) pandemic, telephone and video visits have become increasingly useful. The success of these visits makes a compelling argument for their use during pandemics and by people who live far away from their physicians or are unable to travel for care [3]. The latter is especially relevant for patients suffering from neck pain, as a trip to the clinic could be a struggle. These patients may have difficulty turning their heads while driving [4] or may be taking medications that can cause drowsiness, making it difficult or dangerous to travel [5].

The goal of this article is to discuss how patients with neck pain can be evaluated using telephone and video visits and the specific details that need to be considered during these visits. While limitations exist, we believe that the use of telecommunication technologies to accurately evaluate and diagnose patients is possible.

Telecommunication and security technologies have become so advanced that it is possible to perform telephone and video visits within the patient portals that are tied to health care institutions [6]. In all likelihood, these portals will be widely used in the future, even after the end of the COVID-19 pandemic. The billing and coding for these visits and payment by insurance will also likely improve [7]. As such, the skills needed to hold telephone and video visits should be taught to physician learners as the way of the future, rather than as a momentary need due to the current pandemic.

Neck conditions lend themselves to telehealth evaluation, as their etiologies are often more straightforward than those of conditions that affect other joints. Additionally, red flags are easily identified through verbal history taking, and physical examination of the cervical spine by video is easier than that of smaller joints, such as the hands and wrists, or of more complex joints, like the shoulder.

As the availability of mobile applications increases, musculoskeletal evaluation by telemedicine will become accessible to a greater number of patients. The ability to connect with physicians through popular devices, such as a telephone or tablet, should only serve as an impetus to make telephone and video visits standard practice. This is likely to become a widely-used method for many patients with pain and disability, whether stemming from the cervical spine or from another source.

## Review

Evaluation of neck pain via telephone visit

As an actual physical examination cannot be done by telephone, each question posed by the physician should be aimed at uncovering a suspected injury or condition in order to narrow the differential diagnosis quickly and concisely. Furthermore, each response should be taken in the context of the patient’s history. Table 1 provides questions and instructions that can be given by telephone, examples of responses that patients may give, and the possible diagnoses associated with those responses.

**Table 1:** Cervical spine telephone evaluation questions, responses, and diagnoses.

Question	Possible response	Possible Diagnoses
“Looking at your shoulders in a mirror, do you see any difference between the left shoulder and the right shoulder?”	Affirmative response, stating which shoulder looks higher	Neck muscle spasm; massive rotator cuff tear; neck soft tissue mass; thoracic scoliosis; cranial nerve XI injury
“Looking at your neck in a mirror, do you see any bumps or skin changes?”	Affirmative response, stating where the bump/discoloration is located	Neck muscle spasm; mass; skin tumor; lipoma
“When you sit comfortably, does your head face upward, downward, or straight ahead? Does it favor looking to the right or left?”	Affirmative response, stating which direction the head faces	Poor cervical spine posture
“Do you notice any sunken, swollen, bruised, or red areas on your shoulder? Has anyone else noticed such areas?”	Affirmative response to 1 or more	Areas of atrophy (sunken areas); injury or infection (swelling, bruising, or erythema)
“Does it hurt to press along the back of your neck, near the middle?”	Affirmative response	Cervical sprain; spinous process injury
“Does it hurt to press along the sides of your neck?”	Affirmative response	Paraspinal muscle or trapezius spasm; facet arthropathy
“Does it hurt to press along the sides of your neck near the front?”	Affirmative response	Sternocleidomastoid spasm; anterior scalene spasm; swollen tender lymph nodes
“Are you able to touch your chin to your chest?”	Negative response	Posterior muscle spasm; osteoarthritis
“Are you able to fully extend your neck/look straight up to the sky?”	Negative response	Anterior muscle spasm; osteoarthritis
“Are you able to look fully to the right/left?”	Negative response	Anterior/posterior muscle spasm; osteoarthritis
“Are you able to fully bend your neck, nearly touching your right/left ear to your shoulder?”	Negative response	Anterior/posterior muscle spasm; osteoarthritis
“Do you have any limitations with shoulder motion?”	Affirmative response	Cervical radiculopathy; shoulder injury
“Are you able to shrug both shoulders at the same time?”	Limited bilaterally, or better on 1 side	Trapezius injury; injury to spinal accessory nerve
“Do you have any numbness or tingling in your arms or hands?”	Affirmative response describing where the sensation is occurring	Cervical radiculopathy
“Are you able to give a thumbs-up, make an “OK” sign, and cross your fingers?”	Response of no or limited ability to perform 1 or more	Cervical radiculopathy: radial nerve (thumbs-up); median nerve (“OK” sign); ulnar nerve (crossed fingers)

Evaluation of neck pain via video visit

**Figure 1:**
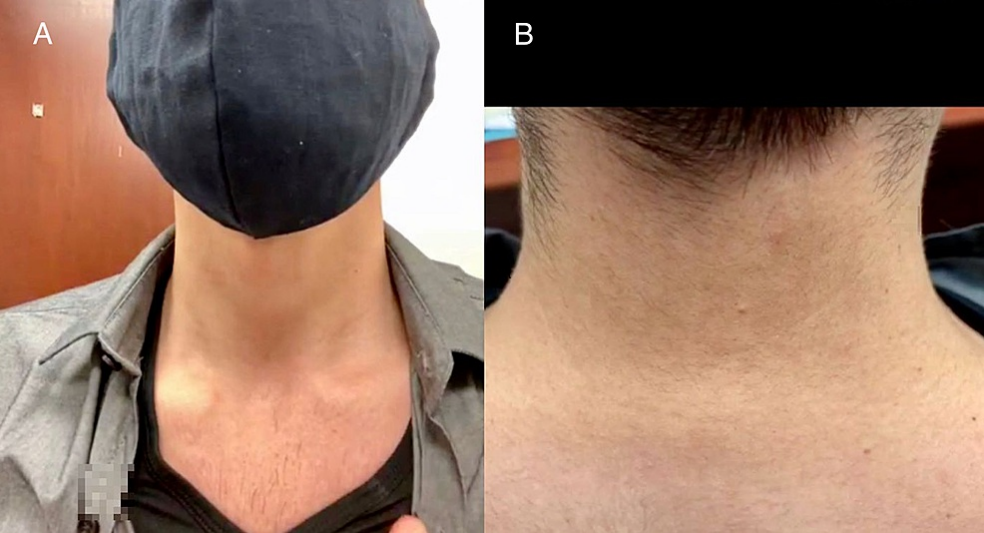
Cervical spine inspection: anterior musculature (A); posterior paraspinal musculature and midline (B).

The neck and shoulders should be examined for asymmetry. The examiner should pay attention to the patient’s head position in their natural neutral position. The patient should be asked to palpate around the neck: the patient can palpate the posterior midline (i.e., the spinous processes), posterior paraspinal musculature, and anterior musculature (Figure 1). The examiner can demonstrate the desired palpation or share an illustration with the patient. With guidance, the patient can report if they notice any cervical lymphadenopathy, bony tenderness, a step-off during palpation of the spinous processes, or tenderness or spasm during palpation of the paraspinous muscles.

**Figure 2:**
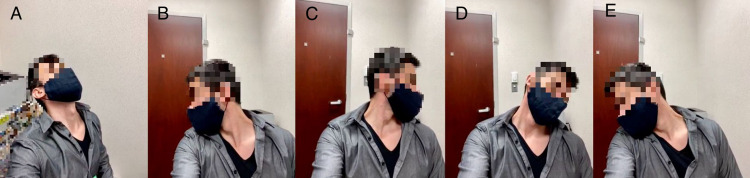
Cervical spine range of motion: extension (A); right (B) and left (C) side rotation; left (D) and right (E) side-bending.

Range of motion tests should be performed next, and these can be quite easily estimated via video. Normal cervical flexion is about 50°; extension, 80°; right and left side bending, 45°; and right and left rotation, 85° (Figure 2). 

**Figure 3:**
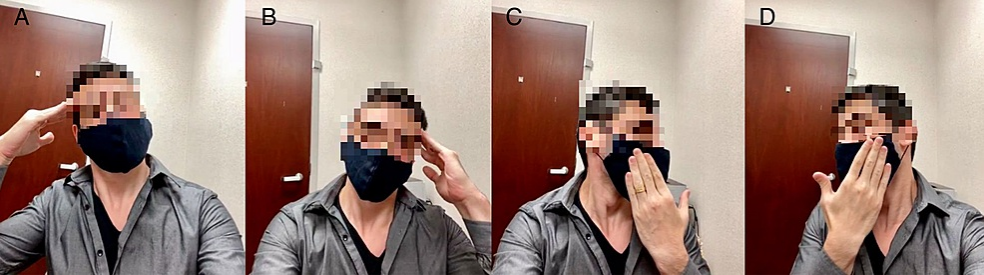
Cervical motion resistance: right (A) and left (B) side-bending; right (C) and left (D) side-rotation.

Testing the entire neck range of motion (i.e., flexion, extension, side bending, and rotation) is important to avoid missing important conditions. The patient can be asked to place their own hand against their head to resist motion and to assess if this causes pain (Figure 3). This maneuver can be used to assess if cervical motion causes pain when against a resistant force.

**Figure 4:**
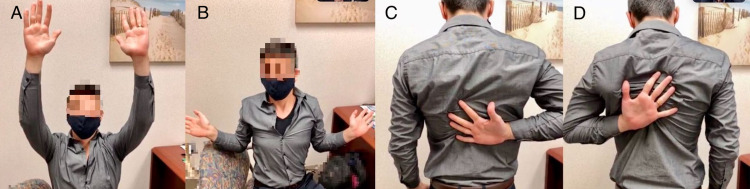
Shoulder range of motion: forward flexion (A); external rotation (B); right (C) and left (D) internal rotation.

Shoulder range of motion should also be evaluated (Figure 4).

**Figure 5:**
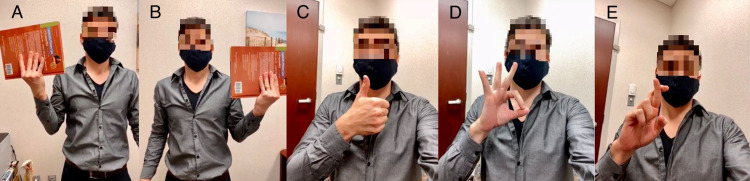
Motor strength. Ask the patient to lift a weight with both arms, reporting differences or pain (A and B). Distal motor strength can be assessed by asking the patient to give a thumbs-up (C), make an “OK” sign (D), and cross their first and second fingers (E).

Strength can be assessed by asking the patient to lift a weight, such as a dumbbell or a book, while they describe any ensuing pain or differences beyond what would be expected for hand dominance (Figure 5). The patient can be asked to give a thumbs-up, make an “OK” sign with their first finger and thumb, and cross their first and second fingers, to assess distal muscle function (Figure 5).

**Figure 6:**
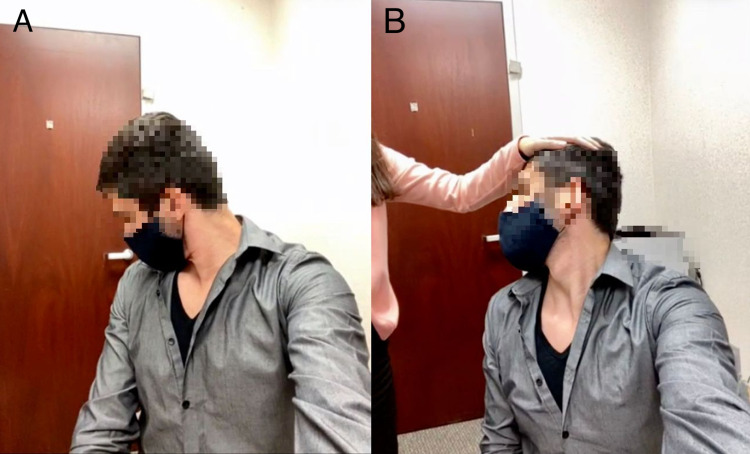
Signs of cervical nerve root irritation or impingement. Cervical nerve root irritation can be assessed by performing the forward flexion test (A) and Spurling test (B).

Signs of cervical nerve root irritation or impingement can be obtained with a forward flexion test: pain will be elicited when the patient is asked to turn their head to the side and then flex their neck forward (Figure 6A). The patient can also be asked to turn and tilt their head backward toward the affected side to mimic an atlantoaxial compression test (Spurling test), or they can ask someone to apply an axial load to the top of their head while their neck is twisted (Figure 6B). Reproduction of radicular pain to the shoulder and arm may suggest cervical nerve root irritation.

Telephone and video visits should be considered important options for the evaluation of musculoskeletal conditions, such as neck pain. Patients with neck pain may have difficulty turning their heads while driving or may be taking medications related to previous neck pain episodes that may cause drowsiness, making it difficult or dangerous for them to travel or operate motor vehicles, and making virtual evaluation a valuable option. Previous literature has reported the use of telehealth visits prior to the COVID-19 pandemic for patients living in rural areas and for those who are older, blind, or disabled [8].

## Conclusions

While telephone and video visits have their limitations, they can allow for the evaluation and accurate diagnosis of patients with neck pain. Questions and instructions can be given by telephone, and patient responses can give clinicians an idea of the etiology of the neck pain. Guided palpation is important, as is the patient’s ability to perform certain movements, such as the thumbs-up or “OK” signs. Telephone and video visits may become valuable options for many patients with pain and disability, whether stemming from the cervical spine or from another source. The skill of managing telephone and video visits should be taught to physician learners as alternatives for future encounters. Scheduling these visits will likely continue, even after the resolution of the COVID-19 pandemic.

## References

[1] Rubin DI (2007). Epidemiology and risk factors for spine pain. Neurol Clin.

[2] Friedman MH, Nelson AJ Jr (1996). Head and neck pain review: traditional and new perspectives. J Orthop Sports Phys Ther.

[3] (2020). Lessons from CEOs: health care leaders nationwide respond to the Covid-19 crisis. NEJM Catal Innov Care Deliv.

[4] Ariëns GA, van Mechelen W, Bongers PM, Bouter LM, van der Wal G (2000). Physical risk factors for neck pain. Scand J Work Environ Health.

[5] Guzman J, Haldeman S, Carroll LJ (2008). Clinical practice implications of the Bone and Joint Decade 2000-2010 Task Force on Neck Pain and Its Associated Disorders: from concepts and findings to recommendations. Eur Spine J.

[6] North F, Crane SJ, Chaudhry R, Ebbert JO, Ytterberg K, Tulledge-Scheitel SM, Stroebel RJ (2014). Impact of patient portal secure messages and electronic visits on adult primary care office visits. Telemed J E Health.

[7] Brown NA (2006). State Medicaid and private payer reimbursement for telemedicine: an overview. J Telemed Telecare.

[8] Douglas MD, Xu J, Heggs A, Wrenn G, Mack DH, Rust G (2017). Assessing telemedicine utilization by using Medicaid claims data. Psychiatr Serv.

